# Asymmetric Inter‐Hemisphere Communication Contributes to Speech Acquisition of Toddlers with Cochlear Implants

**DOI:** 10.1002/advs.202309194

**Published:** 2025-03-31

**Authors:** Xue Zhao, Meiyun Wu, Haotian Liu, Yuyang Wang, Zhikai Zhang, Yuhe Liu, Yu‐Xuan Zhang

**Affiliations:** ^1^ State Key Laboratory of Cognitive Neuroscience and Learning Beijing Normal University Beijing 100875 China; ^2^ Department of Otolaryngology Head and Neck Surgery West China Hospital of Sichuan University Chengdu 610041 China; ^3^ Department of Otolaryngology Head and Neck Surgery Hunan Provincial People's Hospital (First Affiliated Hospital of Hunan Normal University) Changsha 410005 China; ^4^ Department of Otolaryngology Head and Neck Surgery Beijing Chao‐Yang Hospital Capital Medical University Beijing 100025 China; ^5^ Department of Otolaryngology Head and Neck Surgery Beijing Friendship Hospital Capital Medical University Beijing 100050 China

**Keywords:** brain imaging, cochlear implantation, language development, machine learning, speech perception

## Abstract

How the lateralized language network and its functions emerge with early auditory experiences remains largely unknown. Here, early auditory development is examined using repeated optical imaging for cochlear implanted (CI) toddlers with congenital deafness from onset of restored hearing to around one year of CI hearing experiences. Machine learning models are constructed to resolve how functional organization of the bilateral language network and its sound processing support the CI children's post‐implantation development of auditory and verbal communication skills. Behavioral improvement is predictable by cortical processing as well as by network organization changes, with the highest classification accuracy of 81.57%. For cortical processing, behavioral prediction is better for the left than the right hemisphere and for speech than non‐speech processing. For network organization, the best prediction is obtained for resting state, with greater contribution from inter‐hemisphere connections between non‐homologous regions than from within‐hemisphere connections. Most interestingly, systematic connectivity‐to‐activity models reveal that speech processing of the left language network is developmentally supported largely by global network organization, particularly asymmetric inter‐hemisphere communication, rather than functional segregation of local network. These findings collectively confirm the importance of asymmetric inter‐hemisphere communication in formation of the lateralized language network and its functional development with early auditory experiences.

## Graphical abstract

The present study constructs machine‐learning models to resolve developmental relationships among auditory performance, auditory cortical processing, and functional connectivity of the bilateral language network during the first year of restored hearing. The results demonstrate that asymmetric inter‐hemisphere communication contributes significantly to auditory and speech development in pediatric CI users with early auditory experiences.

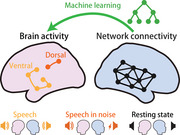

## 1 Introduction

The mature human brain perceives speech via a left‐dominated frontotemporal network.^[^ ^1^, ^2^ ^]^ How this network and its functions emerge in early life remains a fundamental question of language neuroscience. Brain imaging studies with fetuses and infants revealed adultlike organization of functional networks very early in life.^[^ ^3^, ^4^, ^5^ ^]^ For example, functional near‐infrared spectroscopy (fNIRS) detected lateralized auditory and speech responses in the temporal and frontal language areas of the preterm neonate brain at 28‐ to 32‐week gestational age, when cortical organization and neuronal migration are not yet complete.^[^ ^6^ ^]^ Theoretically, emergence of lateralized speech processing requires asymmetric functional organization and information communication between the two hemispheres. However, developmental contribution of network organization, particularly asymmetric inter‐hemisphere communication, is rarely examined. To our awareness, the only related evidence originates from studies showing a decrease in inter‐hemisphere synchrony after around one year of age, which has been proposed to correlate with future language outcomes.^[^ ^7^ ^]^ This phenomenon seems to be mediated by an increase in asymmetric inter‐hemisphere communication.

Here we examine formation of the lateralized language network and its functional development for cochlear implanted (CI) toddlers, with particular emphasis on the role of asymmetric inter‐hemisphere communication. While early auditory experiences are critical for shaping cortical organization,^[^ ^8^ ^]^ the human cortex is capable of responding to sounds as early as ≈20 weeks of gestation,^[^ ^9^ ^]^ rendering the beginning stage of auditory development very hard to assess. Young children with severe to profound sensorineural hearing loss are typically unable to develop auditory and verbal communication skills. Cochlear implantation in the sensitive period for speech acquisition provides these children an opportunity to regain hearing and verbal language,^[^ ^10^ ^]^ allowing us to study the early stages of their auditory and language development. The bilateral language network of 50 congenitally deaf children was examined longitudinally from CI activation using fNIRS, which is used to measure the hemodynamic changes in cortical surface.^[^ ^11^ ^]^ Compared with other neuroimaging methods such as functional magnetic resonance imaging (fMRI), fNIRS is compatible with implant devices, and more tolerable to head movements of toddlers.^[^ ^12^ ^]^ Network development was evaluated by changes in sound evoked activity and functional connectivity between two repeated fNIRS assessments completed within approximately one year of CI experiences. Behavioral outcomes of cochlear implantation were evaluated using three questionnaires in accordance with clinical standards: the Infant Meaningful Auditory Integration Scale (IT‐MAIS/MAIS),^[^ ^13^ ^]^ the Categories of Auditory Performance (CAP)^[^ ^14^ ^]^ and the Speech Intelligibility Rating (SIR),^[^ ^15^ ^]^ which focused on auditory and verbal communication skills. The current study aims to elucidate how the language network development supports behavioral improvements of the CI toddlers.

The high dimensionality of language network data presents challenges for conventional statistical analyses of brain‐behavioral relationships, thereby elevating the risk of false positives arising from numerous statistical comparisons.^[^ ^16^ ^]^ That is why previous studies of language network development are typically limited to a few selected regions and their functional connections.^[^ ^7^, ^17^ ^]^ Instead, machine learning approaches consider numerous features in combination and effectively handle high‐dimensional data.^[^ ^18^ ^]^ This capability is particularly desirable for analyzing on brain‐behavior relationships.^[^ ^19^, ^20^, ^21^ ^]^ Note that conventional machine learning algorithms raise the risk of overfitting, particularly when the dataset is of relatively small size. Refined methods such as nested cross‐validation have been developed to reduce such risks and to promote generalization of machine learning results.^[^ ^22^ ^]^

In this study, machine learning models with the nested cross‐validation framework were used to explain developmental contributions of auditory cortical processing (i.e., Expt. 1) and functional organization of the language network (i.e., Expt. 2) in speech and verbal communication performance during the first year of hearing experiences with CI. Moreover, by using a novel approach, machine learning algorithms were applied to investigate how the functional organization of language network contributes to the emergence of lateralized speech processing in pediatric CI users (i.e., Expt. 3).

## 2 Results

### 2.1 Developmental Contributions of Cortical Processing to Auditory Performance (Expt. 1)

**Figure 1:**
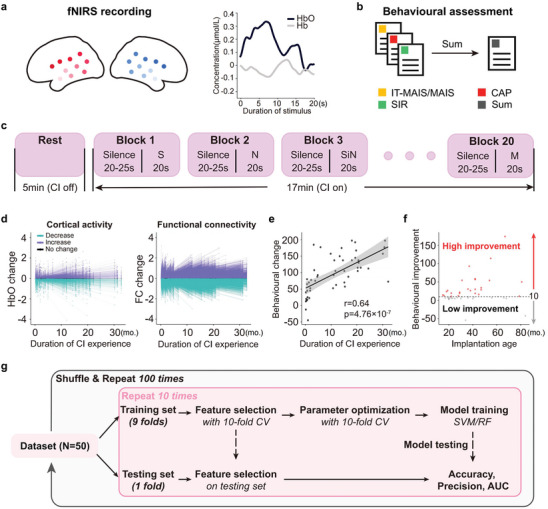
Experimental procedure. a) Left: Cortical location of the functional near‐infrared spectroscopy (fNIRS) channels, with regions ordered from the temporal lobe to the frontal lobe in increasing darkness. Right: An example of sound evoked hemodynamic responses (oxy‐haemoglobin, HbO, and haemoglobin, Hb). b) Assessment of auditory performance of cochlear implanted (CI) children using three questionnaires: the Infant Meaningful Auditory Integration Scale (IT‐MAIS/MAIS), the Categories of Auditory Performance (CAP) and the Speech Intelligibility Rating (SIR). To provide a unified measure for auditory and speech ability, standard scores from these questionnaires were all rescaled to 0–100 and then summed up. c) Time line of fNIRS recording protocol. Each fNIRS recording started with 5 min of resting, during which cochlear implants and hearing aids if in wearing were turned off, followed by ≈17 min of passive listening, during which blocks of 20 s sound presentation were pseudo‐randomly mixed with silence intervals of 20 to 25 s. Four types of sound stimulation (S: natural speech; N: babble noise; SiN: speech in spatially separate noise; M: instrumental music) were presented from two speakers placed 45° to the left and right of the direction that the participant was facing at 65 dB SPL. d) Change between two repeated fNIRS tests for cortical activity (HbO; left panel, 20 channels × 4 conditions × 50 participants = 4000 lines) and functional connectivity (FC; right panel, 190 connections × 5 states × 50 participants = 47 500 lines). All values were normalized to the score of the first test. Developmental changes include both increase (purple) and decrease (green) of activity or connectivity, denoted as colored lines when beyond 95% confidence interval of grand average. e) Change of auditory and speech performance score with increasing CI experiences. There was a significant positive correlation between duration of CI wearing and increase of performance score (r = 0.64, p = 4.76 × 10^−7^). f) Division of CI children into two subgroups (high vs low improvement) based on the median behavioral improvement score, calculated as change of performance score divided by CI duration. g) A nested 10‐fold cross‐validation procedure of machine learning used in the current study.^[^ ^23^ ^]^ Both linear (SVM) and non‐linear (RF) algorithms were applied. Model performance was evaluated using 10‐fold cross‐validation (CV) shuffled and repeated 100 times.^[^ ^21^ ^]^ AUC: area under the curve of the receiver operating characteristic curve.

To predict auditory and speech perception improvement of CI children using fNIRS data (**Figure** **1**a–c), we constructed two types of machine learning models, using random forest (RF) and support vector machine (SVM) algorithms, respectively, with a nested 10‐fold cross‐validation procedure (see Figure 1g and Methods for details). Changes of hemodynamic responses (Figure 1d), divided by between‐test time interval, to four sound conditions within approximately one year of CI experiences were entered as predictors. Post‐CI auditory and speech performance measured using three standard clinical questionnaires improved between two repeated tests (the first and last valid tests after CI implantation), with significant correlation to the duration of CI experiences in between (Figure 1e). Consequently, CI children were divided into two groups according to their median behavioral improvement (Figure 1f), calculated as between‐test score changes divided by the lapsing time interval.

**Figure 2:**
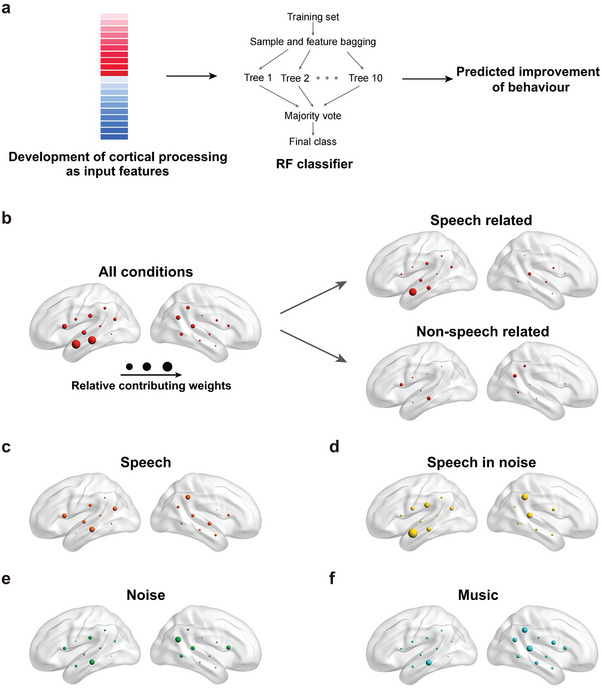
Mean contributing weight distribution across fNIRS channels of random forest (RF) activity‐to‐behavior models. a) Model illustration. b) Contribution of cortical activity when responses to all four sound conditions were entered as model input. Contributing weights of speech related (speech and speech in noise, top) and non‐speech related (noise and music, bottom) conditions were separately presented. c–f) Contributing weights of individual sound conditions. For each panel, mean contributing weights of each channel were evaluated using the Gini importance of the selected fNIRS channels in all 1000 (100 repeated × 10 folds) folds (see Methods for details). A bigger circle represents a higher contributing weight to classification.

The RF models yielded classification accuracy of 76.31%, precision of 80.50% and area under the curve of the receiver operating characteristic curve (AUC) of 82.58% (**Figure** **2**a; Table S1, Supporting Information), similar to machine learning model performance previously reported using similar processing steps and evaluation procedures for CI children using pre‐surgical structural magnetic resonance imaging data.^[^ ^23^ ^]^ The SVM models yielded classification accuracy of 64.98%, precision of 61.11% and AUC of 59.55% (Table S2, Supporting Information). Demographic and audiological factors including age of implantation, gender, time of the first test, ear of implantation, residual hearing of both ears, hearing aid experiences showed at best moderate predictability of behavioral improvement (RF model accuracy: 50.23%; SVM model accuracy: 47.35%). Critically, these factors failed to improve model performance when added to cortical changes (RF model accuracy: 73.82%; SVM model accuracy: 61.33%), with actually slight decrease of performance, indicating that the behavioral predictability of demographic factors, if present at all, was fully absorbed by cortical changes. Thus, we left out these factors from subsequent models.

**Figure 3:**
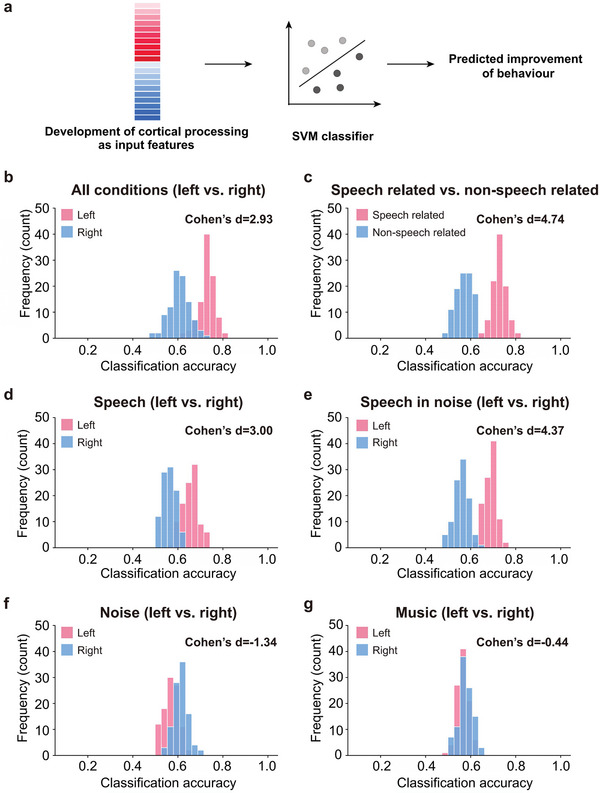
Classification accuracy of support vector machine (SVM) activity‐to‐behavior models. a) Model illustration. b) Classification accuracy of left (pink) and right (blue) hemisphere responses to all four conditions (speech, speech in noise, noise, and music). c) Classification accuracy of all recording channels to speech related (speech and speech in noise) and non‐speech related (noise and music) conditions. d–g) Classification accuracy of left (pink) and right (blue) hemisphere responses to individual sound conditions. Each histogram of classification accuracy was calculated from 100 repetitions of the 10‐fold nested cross‐validation procedure. Cohen's d representing normalized distance of the two colored distributions was presented for each panel, and a higher value indicates a greater difference.

We first compared inter‐hemisphere asymmetry in developmental contributions of cortical processing to auditory performance, as the normal adult brain shows left advantage for speech processing and right advantage for non‐speech processing.^[^ ^2^ ^]^ Using all four sound conditions, cortical development of the left hemisphere shows greater contributing power than the right hemisphere (RF model: Figure 2b, left: 1.28, right: 0.81; SVM model: Table S2, Supporting Information, left: 7.27, right: 6.88). The predictive power of the two hemispheres was also quantitatively compared using SVM models (**Figure** **3**a), revealing higher classification accuracy for the left than the right hemisphere (Figure 3b, *t_(186)_ = 20.72, p = 3.16 × 10^−50^, Cohen's d = 2.93*). Specifically, classification accuracy, precision, and AUC were 72.93% versus 60.73%, 76.62% versus 50.83%, and 72.49% versus 51.17% for the left and right hemispheres (Table S3, Supporting Information). Therefore, both linear (SVM) and non‐linear (RF) machine learning algorithms revealed higher behavioral predictability of the left than right hemisphere.

We next compared contributions of cortical development of speech related and non‐speech related processing. For the previous RF model with all four conditions (Figure 2b), responses to speech related conditions (speech and speech in noise, weight: 1.24) showed higher contributing weight than responses to non‐speech conditions (noise and music, weight: 0.85). This result was quantitatively confirmed using SVM models comparing classification accuracy of speech related and non‐speech related processing (Figure 3c, *t_(198)_ = 33.52, p = 4.10 × 10^−83^, Cohen's d = 4.74*, Table S3, Supporting Information). Notably, the left anterior temporal lobe (aTL), the greatest contributing region in the overall model, remained so only for speech related conditions, suggesting its behavioral contribution is speech specific (Figure 2b).

Behavioral contributions of individual conditions were further examined using both RF (Figure 2; Tables S1 and S4, Supporting Information) and SVM (Figure 3; Tables S2 and S3), Supporting Information models. For speech, there was a left‐hemisphere advantage in both contributing weight (Figure 2c, left: 0.84, right: 0.75) and classification accuracy (Figure 3d, left: 65.59%, right: 55.98%; *t_(198)_ = 21.21, p = 4.45 × 10^−52^, Cohen's d = 3.00*). For speech in noise (SiN), the left advantage was even greater in contribution (Figure 2d, left: 1.05, right: 0.78) as well as accuracy (Figure 3e, left: 68.71%, right: 55.74%; *t_(198)_ = 30.89, p = 1.59 × 10^−76^, Cohen's d = 4.37*), with the most prominent contribution of left aTL (Figure 2d, Speech in noise). In contrast, noise processing showed a right‐hemisphere advantage both in contributing weight (Figure 2e, left: 0.74, right: 0.81) and accuracy (Figure 3f, left: 57.03%, right: 61.26%; *t_(198)_ = −9.52, p = 6.17 × 10^−18^, Cohen's d = −1.34*), demonstrating the right‐hemisphere dominance of non‐speech processing already emerged during early CI hearing experiences. Music processing also showed a right advantage in terms of contribution weight (Figure 2f, left: 0.71, right: 1.15), but not in terms of accuracy (Figure 3g, left: 56.45%, right: 57.68%; *t_(198)_ = −3.11, p = 0.002, Cohen's d = −0.44*).

Overall, SVM and RF algorithms generally revealed left hemisphere advantage for development of speech processing and right hemisphere advantage for development of non‐speech processing in terms of predicting behavioral improvement. Compared to SVM, RF algorithm achieved higher classification performance (Tables S1 and S4, Supporting Information), probably due to its ability to handle highly non‐linear relations among the data.^[^ ^24^ ^]^ Thus, RF algorithm was chosen for the following analyses.

### 2.2 Developmental Contributions of the Language Network to Auditory Performance (Expt. 2)

**Figure 4:**
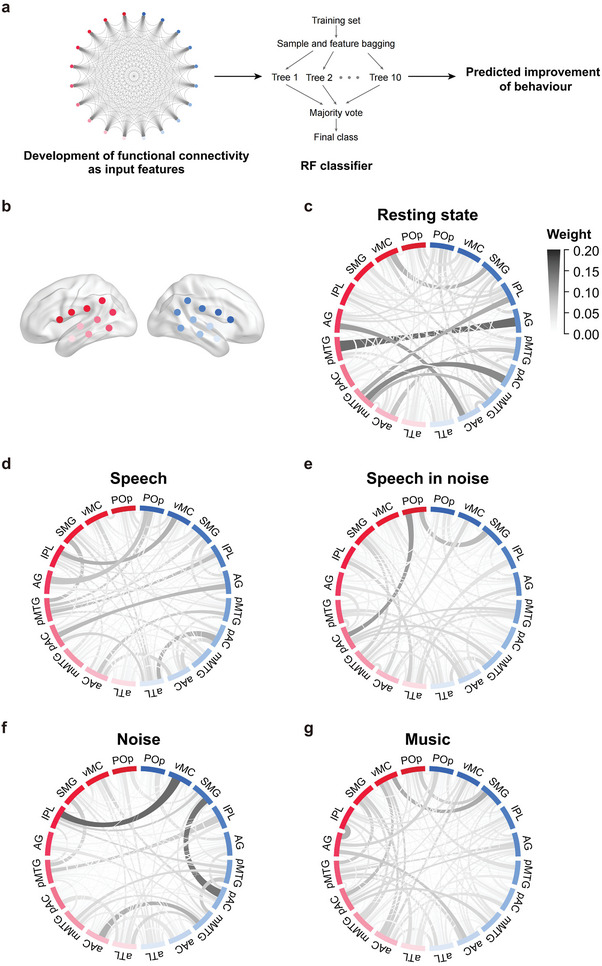
Mean contributing weight distribution of language network connections of random forest (RF) connectivity‐to‐behavior models. a) Model illustration. b) Color coding of fNIRS channels, consistent with Figure 1a. c–g) Mean weight distribution of language network at resting state (c) and passive listening (d: speech, e: speech in noise, f: noise, and g: music). Abbreviations of fNIRS channels: POp, pars opercularis of inferior frontal gyrus; vMC, ventral motor cortex; SMG, supramarginal gyrus; IPL, inferior parietal lobule; AG, angular gyrus; pMTG, posterior middle temporal gyrus; pAC, posterior auditory cortex; mMTG, middle middle temporal gyrus; aAC, anterior auditory cortex; aTL, anterior temporal lobe.

Brain imaging studies with typically developing children revealed that the language network underwent dramatic development during the early years around speech acquisition.^[^ ^17^ ^]^ To elucidate the developmental contributions of language network to auditory performance preceding speech acquisition, we constructed RF models using development of functional connectivity to predict performance improvement with increasing CI experiences (**Figure** **4**a). Connectivity development for resting state and each of the four sound listening conditions (speech, SiN, noise, and music), also divided by the between‐test time interval, was predictive of behavioral improvement (Table S5, Supporting Information, AUC > 0.82), with a slight advantage of classification performance for resting state. For all five states, the sum weight of 90 non‐homologous inter‐hemisphere connections exceeded that of 90 intra‐hemisphere connections (Table S6, Supporting Information). Further, the highest contributing weights for individual connections were observed mainly between non‐homologous sites of the two hemispheres (Figure 4), supporting a significant role of asymmetric inter‐hemisphere communication in auditory and speech development. The intra‐hemisphere contributions showed condition specific distribution between the two hemispheres, with left advantage for SiN and music, and right advantage for resting state, speech, and noise listening (Table S6, Supporting Information). The similar condition specific pattern was observed for classification accuracy (Table S7, Supporting Information).

The significant contribution of the right hemisphere network to speech development poses an apparent surprise, but actually further demonstrates the importance of inter‐hemisphere communication in mediating the left hemisphere advantage of speech responses demonstrated for CI children (in Expt. 1) and generally observed for the adult brain, possibly by conveying right‐hemisphere processing results to the left hemisphere.^[^ ^2^ ^]^

The overall pattern of developmental contribution of the language network was also highly condition specific (Figure 4). For resting state (Figure 4c), major contributing network connections included those between left posterior middle temporal gyrus (pMTG) and right angular gyrus (AG), and from the right auditory cortex to the left higher processing regions (right posterior auditory cortex or pAC to left middle middle temporal gyrus or mMTG and right anterior auditory cortex or aAC to left AG). For speech (Figure 4d), contribution weights were more equally distributed among the bilateral language network, including those from left pAC to right AG, and from the left temporal‐parietal junction areas and the right frontal language region (such as right pars opercularis or POp in the inferior gyrus to left AG/pMTG, right ventral motor cortex or vMC to left inferior parietal lobule or IPL). For SiN (Figure 4e), the contribution of the left POp was most prominent, particularly its connections with the left pAC and the right supramarginal gyrus (SMG). For noise (Figure 4f), there was marked contribution from the right hemisphere (right pAC to right SMG) as well as from inter‐hemisphere connections (left IPL to right vMC). For music (Figure 4g), developmental contributions showed less differential distribution across the language network, but with a greater emphasis on connections within the left hemisphere.

### 2.3 Developmental Contributions of Functional Connectivity to Auditory Cortical Processing (Expt. 3)

**Figure 5:**
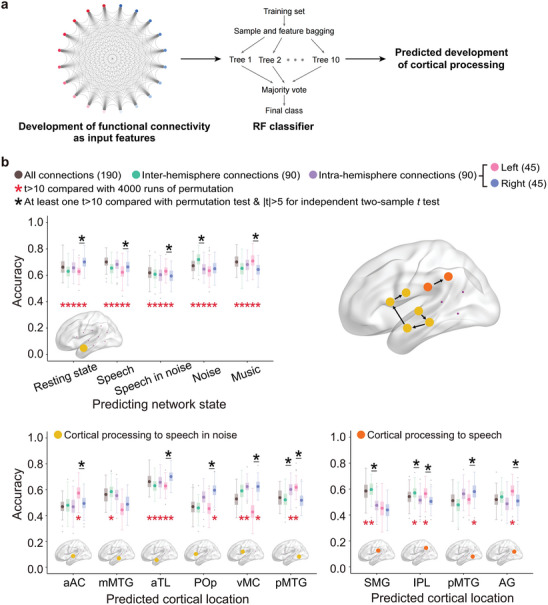
Classification accuracy of random forest (RF) connectivity‐to‐activity models. a) Model illustration. The input feature was comprised of 190 connections in total, including 90 intra‐hemisphere connections (45 in each hemisphere), 90 inter‐hemisphere connections between non‐homologous locations, and 10 inter‐hemisphere connections between homologous locations. b) Top left panel: Successful prediction of speech‐in‐noise processing development at left aTL by functional connectivity changes of the language network at resting and listening (speech, speech in noise, noise, and music) states. Bottom panels: Successful prediction of speech in noise (bottom left) and speech (bottom right) processing development in the left hemisphere by the resting language network. Top right: An overview of the successfully predicted cortical pathway for speech (orange) and speech‐in‐noise (yellow) processing in the left hemisphere. Red stars mark significantly higher classification accuracy than 4000 runs of permutation at *t* > 10; Black stars over horizontal lines indicate significant difference of model performance at |*t*| > 5, which was only compared between models with equal number of input connections, with at least one of the two models yielding successful prediction. POp, pars opercularis of inferior frontal gyrus; vMC, ventral motor cortex; SMG, supramarginal gyrus; IPL, inferior parietal lobule; AG, angular gyrus; pMTG, posterior middle temporal gyrus; mMTG, middle middle temporal gyrus; aAC, anterior auditory cortex; aTL, anterior temporal lobe.

The demonstration of significant contributions of both sound evoked activity and functional connectivity to post‐CI auditory development begs the question of whether network formation relates to auditory processing development, which is a relationship we investigated using machine learning models (**Figure** **5**a).

RF models were first constructed to predict development of SiN processing of the left aTL, which showed the highest contribution to behavioral improvement (Figure 2b,d). SiN processing development at left aTL was predictable, at the criteria of *t* > 10 compared with 4000 runs of permutation test, by development of functional connectivity using all connections of the language network at resting or listening states (Figure 5b top left panel, Table S8 (Supporting Information) for classification performance). To delineate contributions of asymmetric inter‐hemisphere communication, the language network was divided into two groups of 90 connections: inter‐hemisphere connections between non‐homologous regions and intra‐hemisphere connections in both hemispheres. Notably, the predictability of inter‐hemisphere network development was similar to, and exceeded in the state of noise listening, that of intra‐hemisphere network, at the criteria of |t| = 5 for independent two‐sample *t* test (Figure 5b top left panel, Table S8, Supporting Information), indicating that auditory processing in the left temporal lobe depends on formation of the intra‐hemisphere language networks as well as on their asymmetric communication. Further, new RF models were built to compare predictability of the left and right intra‐hemisphere network development (45 connections in each network) for each functional state. While both intra‐hemisphere networks predicted left aTL processing successfully (Figure 5b top left panel, Table S8, Supporting Information), predictability was significantly different between the two hemispheres except for the noise listening state (at the criteria of |t| = 5 for independent two‐sample *t* test), with right hemisphere advantage for resting and speech listening and left hemisphere advantage for SiN and music listening. The right advantage cases, in which left aTL processing was better predicted by the right hemisphere network, particularly accentuate the importance of asymmetric inter‐hemisphere communication. Contributing weight comparisons revealed similar patterns between inter‐ and intra‐, left and right intra‐hemisphere networks (Table S9, Supporting Information).

As network connectivity at resting has been considered to reflect influence of long‐term experiences rather than specific functions of laboratory tests,^[^ ^25^ ^]^ we next analyzed whether resting network developmentally predicts cortical processing of the left language network for each sound condition (Figure 5 bottom panels, see Figure S1, Supporting Information for full results).

For SiN processing (Figure 5b bottom left), prominent predictability by resting network was observed for the ventral auditory processing stream (at the criteria of *t* > 10 compared with 4000 runs of permutation test), starting at aAC and progressing through mMTG and aTL to the frontal language areas of POp and vMC (Figure 5, locations in yellow in top right panel). Developmental predictability of SiN processing at left aAC was acquired from the left intra‐hemisphere connectivity only (*t_(102)_ = 11.49, p = 4.33 × 10^−20^*), showing pronounced left dominance (left vs right *t* test, *|t_(198)_| = 8.92, p = 3.09 × 10^−16^*). At left mMTG, highest predictability arose from asymmetric inter‐hemisphere connections (*t_(102)_ = 13.29, p = 5.46 × 10^−24^*), similar to predictability of intra‐hemisphere networks (inter‐ vs intra‐ *t* test, *|t_(198)_| = 3.28, p = 0.0012*) that could not be attributed to either hemisphere alone. Starting from left aTL, inter‐hemisphere asymmetry was reversed from left to right advantage (left vs right *t* test, aTL: *|t_(198)_| = 11.12, p = 1.21 × 10^−22^*; POp: *|t_(198)_| = 15.59, p = 2.81 × 10^−36^*; vMC: *|t_(187)_| = 22.78, p = 6.52 × 10^−56^*), suggesting a shift from local to global processing involving growing contribution from the opposite hemisphere. In addition, SiN processing at left pMTG, a location near the temporo‐parietal junction that is often associated with multi‐modal social interaction,^[^ ^26^ ^]^ was also developmentally predictable by the resting language network, mainly by the left hemisphere (left vs right *t* test, *|t_(198)_| = 12.17, p = 8.65 × 10^−26^*).

For speech (Figure 5 bottom right), prominent predictability by network at resting state was observed for the dorsal auditory processing stream, including SMG and IPL (Figure 5, locations in orange in top right panel), as well as for multi‐modal areas including pMTG and AG. Speech processing at left SMG and IPL depended primarily on asymmetric inter‐hemisphere communication (comparison with permutation test, SMG: *t_(102)_ = 15.13, p = 8.03 × 10^−28^*, IPL: *t_(103)_ = 12.11, p = 1.71 × 10^−21^*; inter‐ versus intra‐ *t* test, SMG: *|t_(198)_| = 13.54, p = 5.51 × 10^−30^*, IPL: *|t_(198)_| = 6.65, p = 2.87 × 10^−10^*), with a left advantage emerging at IPL (left vs right *t* test, *|t_(198)_| = 7.41, p = 3.61 × 10^−12^*). Speech processing at multi‐modal areas showed opposite patterns of lateralized contribution, with right advantage at pMTG (left vs right *t* test, *|t_(198)_| = 6.91, p = 6.49 × 10^−11^*) and left advantage at AG (left vs right *t* test, *|t_(198)_| = 8.06, p = 7.26 × 10^−14^*).

For non‐speech processing (music and noise), developmental prediction by the resting language network was observed for both ventral and dorsal streams, with inter‐hemisphere asymmetry varied across locations (Figure S1, Supporting Information). For example, music processing at left aAC, early section of the ventral stream, was better predicted by the left‐hemisphere network (left vs right *t* test, *|t_(198)_| = 13.12, p = 1.07 × 10^−28^*), while that at left pAC, early section of the dorsal stream, was better predicted by the right‐hemisphere network (left vs right *t* test, *|t_(198)_| = 15.14, p = 6.47 × 10^−35^*).

## 3 Discussion

Using longitudinal optical neuroimaging data of CI children with prelingual deafness (N = 50), the present study constructed machine‐learning models to resolve developmental relationships among auditory performance, auditory cortical processing, and functional connectivity of the bilateral language network during the first year of restored hearing. First, post‐CI improvement of auditory and verbal communication skills was predicted by development of auditory cortical processing, with a clear advantage for the left over the right hemisphere and for speech over non‐speech stimuli, and the highest contribution came from the left aTL. Further, behavioral improvement was also predictable by functional connectivity development of the language network during resting as well as passive listening states, with greater contribution from asymmetric inter‐hemisphere connections between non‐homologous regions than from within‐hemispheric connections. Most interestingly, speech in noise processing in the left aTL was developmentally predicted by language network connectivity at resting as well as listening states, with similar contributions from asymmetric inter‐hemisphere and within‐hemispheric connections. Systematic connectivity‐to‐activity models revealed that the resting language network developmentally predicted speech in noise processing of the left ventral auditory stream, with similar contribution from asymmetric inter‐hemisphere and within‐hemisphere connections and a right hemisphere advantage. In contrast, speech processing of the dorsal auditory stream was developmental predicted by the resting language network, with prominent contribution from asymmetric inter‐hemisphere communication and a left hemisphere advantage. The three lines of evidence collectively confirmed the importance of asymmetric inter‐hemisphere communication in formation of the lateralized language network and its functional development with early auditory experiences.

### 3.1 Leftward Dominance of Speech Processing Develops Within One Year of Hearing Experiences

Our results demonstrated that during around the first year of CI hearing experiences, the auditory cortex of the CI children underwent functional differentiation, with the left anterior temporal lobe contributing most to behavioral improvement.

Similar functional differentiation has been demonstrated for normal developing brain at birth, as left hemispheric advantage was observed in cortical responses to natural speech in neonates and infants using fNIRS,^[^ ^27^ ^]^ fMRI,^[^ ^28^ ^]^ EEG,^[^ ^29^ ^]^ and optical topography,^[^ ^30^ ^]^ although a few studies also revealed bilateral and right‐lateralized patterns of activation to natural speech.^[^ ^31^, ^32^ ^]^ Overall, it is considered that left dominance for speech processing is already established in neonates.^[^ ^3^ ^]^

Auditory cortex of prelingually deafened children showed little response to speech and right favoring responses to non‐speech sounds upon CI activation.^[^ ^33^ ^]^ Left hemispheric advantage for visual language was observed in CI children (mean age: 8.5 yrs) with ≈5.3‐y CI experiences^[^ ^34^ ^]^ and for deaf children to similar extent with and without CI usage.^[^ ^35^ ^]^ The current results suggest that CI‐mediated auditory experiences allowed the children to develop an advantage to changes in cortical processing in the left hemisphere within around one year, and provide the first direct evidence associating such development of lateralized speech processing with the children's behavioral improvement.

### 3.2 Asymmetric Inter‐Hemisphere Communication Contributes Significantly to Auditory and Speech Development

Using functional connectivity instead of sound evoked activity as input features to predictive machine learning models yielded different contribution patterns of the language network to auditory development. The connectivity model results demonstrate that behavioral improvement of CI children with one year of hearing experiences were driven by multiple non‐homologous inter‐hemisphere and a few intra‐hemisphere functional connections. To our awareness, the current study provides the first piece of evidence on development of non‐homologous inter‐hemisphere connectivity and its behavioral contribution. Homologous inter‐hemisphere connectivity was shown to increase across the third trimester of gestation.^[^ ^36^ ^]^ Infant resting‐state fMRI studies indicated that homologous inter‐hemisphere connectivity for language‐related regions increased from 40‐weeks postmenstrual age through the first year of life, followed by a decrease in the second year of life.^[^ ^7^, ^17^ ^]^ The development of functional connectivity between bilateral IFGs was associated with future language performance,^[^ ^7^ ^]^ assessed at 4 years of age, in that future good performers showed prominent bell‐shaped trajectory, while future poor performers did not. This association was attributed to increase of asymmetric inter‐hemisphere communication that may accompany the decrease of inter‐hemisphere synchrony between homologous regions. The current connectivity results that the highest contributing weights for individual connections were observed mainly between non‐homologous sites of the two hemispheres (Figure 4) provide direct support for this suggestion.

Note that the CI children were a couple of years older than the normal hearing infants of the previous MRI studies. Though both groups were functionally in the developmental stage preceding speech acquisition, the CI children may have undergone more advanced structural development. Even if this were the case, the CI children's language network showed little speech responses upon CI activation,^[^ ^33^ ^]^ functionally inferior to neonates.^[^ ^31^ ^]^ Deafened animal models indicated that their auditory cortex, compared with normal‐hearing peers, underwent an accelerated form of functional development after cochlear implantation.^[^ ^37^ ^]^ Similar to the animal results, the developmental pattern observed for CI children might also reflect an accelerated form of the normal brain at earlier years.

Our results also identified a few intra‐hemisphere connections for significant contributions to post‐CI behavioral improvement, primarily from pMTG to SMG and from pAC to POp in the left frontotemporal network, and pAC to aTL in the right temporal cortex. In the normal hearing brain, intra‐hemisphere functional connectivity in the left frontotemporal language network did not develop significantly from 31 weeks of gestation through the first postnatal month, remaining weak in toddlerhood.^[^ ^17^, ^31^ ^]^ The left frontotemporal language network appears to develop in early childhood, as age‐related increase of left asymmetry was observed for children aged 2–7 years^[^ ^38^ ^]^ and between children of 3 and 5 years old.^[^ ^39^ ^]^ The current study provides the first evidence linking the developing frontotemporal network to improvement of auditory and speech performance. Note that in the context of the dual‐stream theory for auditory and language processing,^[^ ^1^, ^2^, ^40^, ^41^ ^]^ two out of the three major contributing intra‐hemisphere connections lie in the left dorsal stream, and one lies in the right ventral stream. In the adult brain, the dorsal stream is strongly left lateralized, while the ventral stream is more bilateral,^[^ ^1^, ^2^ ^]^ but little is yet known about the development of such inter‐hemisphere asymmetry. The intra‐hemisphere contribution results indicated that early auditory development preceding speech acquisition already reflects the different extents of lateralization of the dorsal and ventral streams in their mature form.

### 3.3 Asymmetric Inter‐Hemisphere Communication Contributes to Emergence of Lateralized Speech Processing

The current study directly tested and confirmed the hypothesis that asymmetric inter‐hemisphere communication drives emergence of lateralized speech processing, demonstrating prominent contributions of functional connections within the right hemisphere and between non‐homologous regions of the two hemispheres to speech processing development in the left hemisphere. The connectivity‐to‐activity contributions provide developmental evidence for the dual‐stream theory of auditory processing based primarily on electrophysiological studies of animal brain and functional imaging studies of the adult human brain,^[^ ^1^, ^42^ ^]^ with a ventral stream present in both hemispheres responsible for sound‐to‐meaning mapping and a dorsal stream dominantly present in the left hemisphere responsible for auditory‐to‐motor mapping and information integration. Consistent with the distinctive roles of the two streams in the mature brain, resting state connectivity developmentally predicts speech‐in‐noise processing of the left ventral stream and speech processing of the left dorsal stream.

These results also provide the first evidence on how functional organization of the language network developmentally supports the dual streams of speech processing. The connectivity‐to‐activity machine learning models revealed that such development depended more on formation of the right‐hemisphere network than on formation of the left‐hemisphere network, demonstrating that the lateralized function arises from inter‐hemisphere coordination of the bilateral language network instead of from functional separation of the local network. In contrast, though speech processing development of the left dorsal stream relied more on formation of the left‐hemisphere network, it also showed greater contribution from asymmetric inter‐hemisphere than within‐hemisphere communication, suggesting dependence on global rather than local network coordination.

In addition, the current study provides an example of using machine learning to resolve relationships between different neural processes. To date, the extensive application of machine learning mostly involves building classification or regression models for neuro‐behavioral relations,^[^ ^19^, ^20^, ^23^, ^43^, ^44^ ^]^ probably due to the central aim of human neuroscience being understanding the neurobiology of cognition and behavior (neuro‐to‐behavior relations). However, another important, and necessary, goal of neuroscience is to elucidate causal chains of neural processes (neuro‐to‐neural relations). To our awareness, this is the first machine learning study using network connectivity to predict cortical processing, providing a novel approach for analyzing neuro‐to‐neural relations in the human brain, the most complex system in the natural world.

## 4 Experimental Section

### Participants

A total of 50 children (19 females, mean age of implantation: 36.80 ± 18.26 months, see Table S10, Supporting Information for details) with severe‐to‐profound prelingual sensorineural hearing loss were recruited from the Peking University First Hospital before cochlear implant (CI) surgery and taken to the auditory cognitive neuroscience laboratory of Beijing Normal University for cortical functional evaluations. All child participants complied with the cochlear implantation criteria set out by China National Guidelines for Cochlear Implantation.^[^ ^45^ ^]^ To participate in the current study, each child had to complete at least two tests after CI with valid cortical functional and behavioral assessments. The Ethical Committee of Peking University and the Peking University First Hospital approved this research protocol (Reference: 2018 research No. 239). Informed consent from the parent(s) or guardian(s) was obtained to use these fNIRS data and children's clinical outcome data for the research.

### fNIRS Recording

Children were assisted in sitting on toddler‐sized chairs or the laps of their guardians, in a sound‐attenuated booth. Continuous fNIRS recordings were conducted using a size 52 cap placed on their head while they watched a muted cartoon video or engaged in silent play with toys. The data were recorded using 4 LED sources, emitting light at wavelengths of 760 and 850 nm, along with 4 detectors (with no short channels) from the NIRSport2 systems developed by NIRx Medical Technologies (USA). Probe placement (Figure S2, Supporting Information) involved a 3 cm probe separation, resulting in a theoretical montage comprising a total of 10 channels in each hemisphere (See Table S11, Supporting Information for Montreal Neurological Institute (MNI) space of the 20 fNIRS channels). The sampling rate was set at 7.81 Hz. Each recording session lasted for 22 min in total, consisting of 5 min of resting state with the CI and hearing aid if in wearing switched off, followed by 17 min of auditory stimulation with the devices turned on.

Sounds were presented using custom programs with Psychtoolbox for MATLAB^[^ ^46^ ^]^ from two cube BOSE loudspeakers placed at 45° left and right to the direction the participant was facing. The sound level was 65 dB SPL measured at the participant's head position. Passive listening consisted of 20 blocks of sound presentation from 4 conditions (speech, noise, speech‐in‐noise, and music) in a pseudo‐randomized order, excluding consecutive blocks of the same sound. Speech stimuli were adapted from audio books of child stories; babble noise was synthesized from multiple story recordings of 6 different speakers; music was selected from soothing instrumental music recordings. For speech in noise condition, speech and noise stimuli were separately presented from the two speakers. Each block lasted for 20 s, followed by a silence interval varying between 20 to 25 s. To relieve the child's physical or mental tiredness, the 20 blocks were divided into three sessions with breaks between sessions. The experiment would stop whenever the participant showed crying, emotional disturbance, frequent head movements, or at the request of the guardian.

### fNIRS Data Processing

Raw data were screened for artifacts caused by head movements using FC_NIRS^[^ ^47^ ^]^ (https://www.nitrc.org/frs/?group_id=883) and custom scripts in MATLAB (The MathWorks, USA). Recordings of a given channel were regarded as invalid and excluded from further analysis if heartbeats (1–1.5 Hz) were not detectable or the coefficient of variation exceeded 20%.^[^ ^48^ ^]^ The valid recordings, with no >30% channels to be excluded.^[^ ^49^ ^]^ They were then preprocessed using the HOMER2 toolbox^[^ ^50^ ^]^ (https://www.nitrc.org/frs/?group_id=619), including motion artifact correction with combination wavelet and spline methods supposed by Lorenzo,^[^ ^51^ ^]^ bandpass filtering of 0.01–0.2 Hz^[^ ^52^ ^]^ to exclude physiological noise such as breathing and drifts, hemodynamic signal calculation, and baseline correction. Cortical locations of the fNIRS channels were mapped using a recent transcranial brain atlas for children^[^ ^53^ ^]^ and a localizing system using child head manikin.^[^ ^54^ ^]^ Auditory cortical responses were measured using oxy‐haemoglobin (HbO) signal, as it is known to be more strongly correlated with the cognitive activity^[^ ^55^, ^56^, ^57^ ^]^ and more sensitive to the regional cerebral blood flow changes.^[^ ^55^, ^58^ ^]^ Signal of the first 5 s after sound onset, reported to be unrelated to cognitive activity, was removed.^[^ ^59^ ^]^ The averaged HbO magnitude over valid blocks with 5–20s time windows was taken as auditory cortical response at each test. Between the two tests, cortical responses of most fNIRS channels and sound conditions increased or decreased (Figure 1d). Functional connectivity was calculated using Pearson correlation analyses. Hemodynamic changes of each valid block were normalized with a mean of 0 and standard deviation of 1 and then were concatenated across blocks to obtain long time series,^[^ ^60^ ^]^ with at least 3 valid blocks (>1 min) for each sound condition. Then, a full Pearson correlation was conducted on time series between pairs of channels to yield a symmetrical 20 × 20 matrix of functional connectivity. The correlation coefficient was transformed into Fisher_Z score. For resting state, functional connectivity was calculated in a similar method from consecutive 120 s of clean data. Between the two tests, most functional connections of the language network showed developmental changes (Figure 1d).

### Auditory and Speech Outcome Measures

Following clinical standard practice, auditory and speech performance of the CI participants was assessed using three questionnaires with caretakers: the Infant Meaningful Auditory Integration Scale (IT‐MAIS/MAIS),^[^ ^13^ ^]^ the Categories of Auditory Performance (CAP),^[^ ^14^ ^]^ and the Speech Intelligibility Rating (SIR).^[^ ^15^ ^]^

Given that speech perception measures were not yet directly obtainable for most CI children, auditory and speech‐perception questionnaires were completed by their guardians based on their daily observations. To provide a unified measure for the participants’ auditory/speech ability, standard scores from these questionnaires were all rescaled to 0–100 and then summed up for each CI child. Post‐CI auditory and speech score measured using sum of three standard clinical questionnaires improved between the two tests, with a significant correlation to the duration of CI experiences in between (Figure 1e and *r = 0.64, p = 4.76 ×* *10^−7^*). To account for variance of CI wearing duration, behavioral improvement was calculated using between‐test score changes divided by the lapsing time interval. The 50 participants were then divided into two subgroups based on the median of behavioral improvement score (Figure 1f). The two groups were comparable in all the demographic and audiological factors, including age of implantation, gender, time of the first test, ear of implantation, residual hearing of both ears, hearing aid experiences (**Table** **1**, Table S10, Supporting Information), except in their improvement score (*p* <0.001).

**Table 1:** Demographic and audiological information for 50 cochlear implanted (CI) child participants.

	Low improvement group Mean [SD]	High improvement group Mean [SD]	Group comparison (*p* value)
Number	25	25	/
Gender (M: F)	18:7	13:12	0.145
CI side (L: R: B)	10:14:1	8:17:0	0.469
First test time after CI (mo.)	0.29 (0.32)	0.20 (0.27)	0.257
CIU (dB)	104.68 (8.67)	103.20 (10.51)	0.590
Non‐CIU (dB)	102.52 (12.66)	97.40 (14.02)	0.182
AoI (mo.)	34.28 (18.56)	39.32 (17.97)	0.334
HAtime (mo.)	15.92 (12.82)	13.76 (10.03)	0.510
Behavioral improvement score	1.90 (15.35)	40.43 (37.87)	<0.001

M, male; F, female; L, left; R, right; B, both; AoI, age of implantation; CIU, residual hearing on CI side; Non‐CIU, residual hearing on non‐CI side; HAtime, duration of hearing aid; Behavioral improvement score = Behavioral change/duration of two tests.

### Multivariate Machine Learning Classification

Both linear (support vector machine, SVM) and non‐linear (random forest, RF) machine learning algorithms were used for classification analyses in this study. SVM has become a popular choice in neuroimaging studies, due to its excellent ability to handle high‐dimensional data and to yield promising results.^[^ ^61^, ^62^, ^63^ ^]^ RF is a nonlinear, tree‐based, and nonparametric machine learning method,^[^ ^64^, ^65^ ^]^ showing robustness to overfitting problems and advantages in handling highly non‐linearly correlated data.^[^ ^24^, ^66^, ^67^, ^68^ ^]^ The scikit‐learn library (version: 0.21.2) was used to implement SVM and RF classification (http://scikit‐learn.org/).^[^ ^69^ ^]^

Neural data from each child were converted into an S‐by‐V matrix, where S is the number of participants and V is the number of features (channels), and the matrix was normalized so that mean = 0 and SD = 1 for each feature.^[^ ^23^ ^]^ A nested 10‐fold cross‐validation (10F‐CV) procedure^[^ ^20^, ^21^, ^23^ ^]^ with three levels (inner, middle, and outer) was applied for feature selection, parameter optimization, and model validation (Figure 1g).

At the inner level, two different feature selection algorithms were employed based on the training set for avoiding overfitting. The first algorithm adopts the support vector machine recursive feature elimination (SVM‐RFE) procedure^[^ ^70^, ^71^ ^]^ to select the most relevant features. Specifically, SVM‐RFE can select an optimal combination of features with the highest classification accuracy by removing the less important features iteratively. By setting different number of features (from 1 to V) with stepwise iteration of adding one feature at each time, there are V optimal combinations of features, among which the combination with the highest classification accuracy was defined as the optimal feature subset. The second algorithm adopts SelectFromModel (SFM)^[^ ^72^ ^]^ to select effective features. Specifically, for a specific threshold set, SFM can calculate an optimal combination of features by removing features whose importance are less than the threshold. Ten thresholds from 0 to “max feature importance” (calculated based on the training set) were applied to generate 10 optimal combinations of features, among which the combination with the highest classification accuracy was defined as the optimal feature subset.

At the middle level, two different classifiers (i.e., SVM with “linear” kernel and RF) were used. For “linear” kernel SVM classifier, models were generated with wide range of C parameter on the selected features, choosing the model parameter “C” with GridSearchCV function based on the training dataset. For RF classifier, default parameters were used.

At the outer level of 10F‐CV, models were built based on the training dataset, using the selected features and optimized parameter (for “linear” kernel SVM classifier), and then applied the trained model to classify the testing dataset in each of outer 10F‐CV. This loop was repeated ten times to test through all participants.

Finally, to ensure the reliability and robustness, the nested 10F‐CV process were repeated 100 times with participants randomly shuffled. Classification accuracy, precision, and area under the curve of the receiver operating characteristic curve (AUC) were calculated using the averaged value of 100 times nested 10F‐CV. AUC quantifies a model's ability to classify, with a larger value indicating a better classification power.^[^ ^20^, ^73^ ^]^

A permutation test was employed to determine whether the classification accuracy was significantly better than chance.^[^ ^19^, ^20^, ^23^ ^]^ To achieve this, the group labels were randomly permuted 4000 times. The mean classification accuracy across 4000 permutation tests converged at 50% (chance level).

### Contributing Weight of Features

In each fold of the outer 10F‐CV, the weight for each feature was obtained based on Gini importance of the RF model,^[^ ^65^, ^74^, ^75^ ^]^ the higher of which the greater contribution of the feature to classification.^[^ ^69^ ^]^ Given the reliability and robustness, 100 times nested 10F‐CV procedure was applied to classify two groups, and feature selection procedure in each fold of the outer 10F‐CV was conducted using a slightly different sample subset, different features were selected in each fold. The relative contributing weight for each feature was calculated by averaging weights in all 1000 (100 × 10) folds.^[^ ^18^, ^20^, ^21^, ^76^ ^]^

### Statistical Analysis

When assessing the significance of an observed distribution of 100 classification accuracies, a null distribution was estimated from 4000 permutations on data with scrambled labels. Comparison of an observed distribution to the null or another observed distribution was conducted using the unpaired, two‐tailed Student's *t*‐test, with the significance criterion set at |*t*| > 10 if the null distribution was included and at |*t*| > 5 if not. All analyses were performed with R (https://www.r‐project.org/).

## Conflict of Interest

The authors declare no conflict of interest.

## Author Contributions

X.Z. and Y.‐X.Z. designed the study and presented the manuscript; X.Z. performed the machine learning analyses; M.W. collected and analyzed neuroimaging data; Y.L., Y.W., Z.Z., and H.L. provided clinical tests and medical care.

## Supporting information



Supporting Information

## Data Availability

The data that support the findings of this study are available on request from the corresponding author. The data are not publicly available due to privacy or ethical restrictions.
