# Supplementary material for: Asymmetric Inter‐Hemisphere Communication Contributes to Speech Acquisition of Toddlers with Cochlear Implants
Source: Adv Sci (Weinh). 2025 Mar 31;12(20):2309194. doi: 10.1002/advs.202309194 (PMC12120705; doi:10.1002/advs.202309194)
Supplement: Supplementary file 1 — Supporting Information [file ADVS-12-2309194-s001.docx]

**Supplementary Information**

**List of Supplementary Figures**

**Figure S1:** Classification accuracy of random forest (RF) connectivity-to-activity models (using development of functional connectivity at resting state to classify development of cortical processing in the left hemisphere).

**Figure S2:** fNIRS montage and channels.

**List of Supplementary Tables**

**Table S1:** Classification performance and relative contributing weights of random forest (RF) activity-to-behaviour models (using development of cortical processing to classify improvement of auditory and speech performance).

**Table S2:** Classification performance and contributing weights of support vector machine (SVM) activity-to-behaviour models (using development of cortical processing to classify improvement of auditory and speech performance).

**Table S3:** Classification performance of support vector machine (SVM) activity-to-behaviour models (using development of cortical processing to classify improvement of auditory and speech performance).

**Table S4:** Classification performance of random forest (RF) activity-to-behaviour models (using development of cortical processing to classify improvement of auditory and speech performance).

**Table S5:** Classification performance of random forest (RF) connectivity-to-behaviour models (using development of language network at resting state to classify improvement of auditory and speech performance).

**Table S6:** Relative contributing weight of language network connections of random forest (RF) connectivity-to-behaviour models (using development of language network at resting state to classify improvement of auditory and speech performance).

**Table S7:** Classification accuracy of random forest (RF) connectivity-to-behaviour models (using development of language network to classify improvement of auditory and speech performance).

**Table S8:** Classification accuracy of random forest (RF) connectivity-to-activity models (using development of language network at resting state and four types of stimulus conditions to classify development of left aTL responses to speech in noise).

**Table S9:** Relative contributing weights of random forest (RF) connectivity-to-activity models (using development of language network at resting state and four types of stimulus conditions to classify development of left aTL responses to speech in noise).

**Table S10:** Demographic and audiological information for 50 cochlear implanted (CI) children.

**Table S11:** Montreal Neurological Institute (MNI) space of the 20 fNIRS channels.

Supplementary Figures


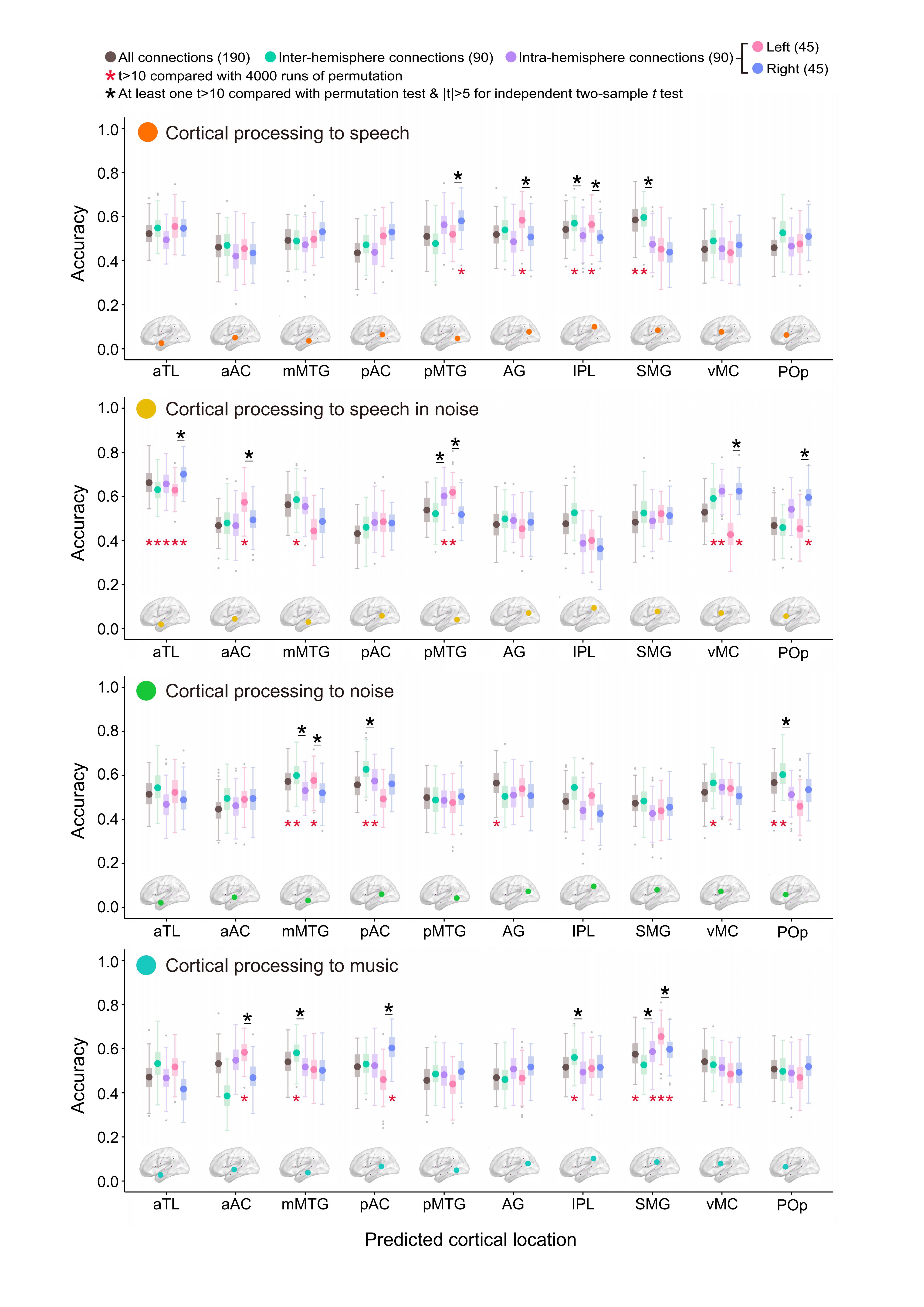


**Figure S1:** Classification accuracy of random forest (RF) connectivity-to-activity models (using development of functional connectivity at resting state to classify development of cortical processing in the left hemisphere).

**Figure S2:
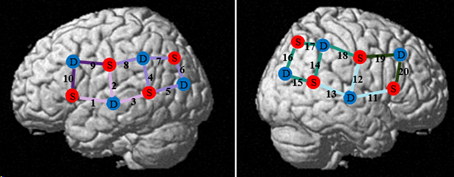
** The theoretical fNIRS montage and channels. D = Detector; S = Source; the numbers in figure indicate the channel number.

Supplementary Tables

**Table S1.** Classification performance and relative contributing weights of random forest (RF) activity-to-behaviour models (using development of cortical processing to classify improvement of auditory and speech performance).

| **Condition** | | Accuracy  (%) | Precision  (%) | AUC  (%) |  | Hemisphere | Weight |
| --- | --- | --- | --- | --- | --- | --- | --- |
| **All conditions** | 76.31 | | 80.50 | 82.58 |  | Left | 1.28 |
|  |  |  |  |  |  | Right | 0.81 |
| **Speech related** | | | | |  |  | 1.24 |
| **Non-speech related** | | | | |  |  | 0.85 |
| **Speech** | 78.59 | | 82.89 | 84.53 |  | Left | 0.84 |
|  |  |  |  |  |  | Right | 0.75 |
| **Speech in noise** | 74.63 | | 78.38 | 80.99 |  | Left | 1.05 |
|  |  |  |  |  |  | Right | 0.78 |
| **Noise** | 73.85 | | 76.74 | 81.52 |  | Left | 0.74 |
|  |  |  |  |  |  | Right | 0.81 |
| **Music** | 71.23 | | 74.33 | 76.23 |  | Left | 0.71 |
|  |  |  |  |  |  | Right | 1.15 |

**Table S2.** Classification performance and contributing weights of support vector machine (SVM) activity-to-behaviour models (using development of cortical processing to classify improvement of auditory and speech performance).

| **Condition** | Accuracy  (%) | Precision  (%) | AUC  (%) |  | Hemisphere | Weight |  |
| --- | --- | --- | --- | --- | --- | --- | --- |
| **All conditions** | 64.98 | 61.11 | 59.55 |  | Left | 7.27 |  |
|  |  |  |  |  | Right | 6.88 |  |
| **Speech related** | | | |  |  | 7.36 |  |
| **Non-speech related** | | | |  |  | 6.79 |  |
| **Speech** | 66.53 | 66.10 | 57.95 |  | Left | 3.46 |  |
|  |  |  |  |  | Right | 2.61 |  |
| **Speech in noise** | 69.83 | 69.47 | 68.24 |  | Left | 3.10 |  |
|  |  |  |  |  | Right | 2.11 |  |
| **Noise** | 60.80 | 51.75 | 44.39 |  | Left | 2.92 |  |
|  |  |  |  |  | Right | 3.22 |  |
| **Music** | 57.30 | 37.45 | 38.91 |  | Left | 3.37 |  |
|  |  |  |  |  | Right | 2.81 |  |

For linear SVM, the weight vector perpendicular to the hyperplane represents the importance of the feature. A higher absolute value of the weight designates a greater contribution of the corresponding feature to the classification.

**Table S3.** Classification performance of support vector machine (SVM) activity-to-behaviour models (using development of cortical processing to classify improvement of auditory and speech performance).

| **Condition** | Hemisphere | Accuracy  (%) | Precision  (%) | AUC  (%) |
| --- | --- | --- | --- | --- |
| **All conditions** | Left | 72.93 | 76.62 | 72.49 |
|  | Right | 60.73 | 50.83 | 51.17 |
| **Speech related** |  | 72.71 | 77.10 | 70.32 |
| **Non-speech related** |  | 57.05 | 38.63 | 34.73 |
| **Speech** | Left | 65.59 | 62.74 | 57.13 |
|  | Right | 55.98 | 34.12 | 40.47 |
| **Speech in noise** | Left | 68.71 | 68.80 | 71.39 |
|  | Right | 55.74 | 31.71 | 45.63 |
| **Noise** | Left | 57.03 | 38.19 | 45.67 |
|  | Right | 61.26 | 55.26 | 51.62 |
| **Music** | Left | 56.45 | 30.50 | 40.29 |
|  | Right | 57.68 | 42.12 | 42.29 |

**Table S4.** Classification performance of random forest (RF) activity-to-behaviour models (using development of cortical processing to classify improvement of auditory and speech performance).

| **Condition** | Hemisphere | Accuracy  (%) | Precision  (%) | AUC  (%) |
| --- | --- | --- | --- | --- |
| **All conditions** | Left | 73.55 | 77.32 | 80.61 |
|  | Right | 73.43 | 76.73 | 82.00 |
| **Speech related** |  | 75.34 | 78.96 | 81.74 |
| **Non-speech related** |  | 72.30 | 76.07 | 78.38 |
| **Speech** | Left | 79.04 | 84.01 | 86.10 |
|  | Right | 75.31 | 79.65 | 81.92 |
| **Speech in noise** | Left | 71.87 | 74.45 | 80.95 |
|  | Right | 76.78 | 81.18 | 79.85 |
| **Noise** | Left | 74.75 | 78.22 | 79.64 |
|  | Right | 74.98 | 77.65 | 83.82 |
| **Music** | Left | 67.83 | 70.70 | 73.15 |
|  | Right | 73.78 | 76.92 | 81.39 |

**Table S5.** Classification performance of random forest (RF) connectivity-to-behaviour models (using development of language network at resting state to classify improvement of auditory and speech performance).

| **Condition** | Accuracy  (%) | Precision  (%) | AUC  (%) |
| --- | --- | --- | --- |
| **Resting state** | 81.57 | 87.36 | 89.18 |
| **Speech** | 75.11 | 78.71 | 82.18 |
| **Speech in noise** | 77.04 | 81.74 | 82.96 |
| **Noise** | 76.72 | 80.00 | 82.83 |
| **Music** | 77.28 | 81.58 | 84.93 |

**Table S6.** Relative contributing weight of language network connections of random forest (RF) connectivity-to-behaviour models (using development of language network at resting state to classify improvement of auditory and speech performance).

| **Condition** | Inter-hemisphere connections | |  | Intra-hemisphere connections | | | | |
| --- | --- | --- | --- | --- | --- | --- | --- | --- |
|  | Sum  (100) | Non-homo  (90) |  | Sum  (90) | Left intra (45) | | Right intra (45) | |
| **Resting state** | 2.39 | 2.15 |  | 1.58 | | 0.65 | | 0.93 |
| **Speech** | 2.55 | 2.27 |  | 1.56 | | 0.71 | | 0.85 |
| **Speech in noise** | 2.02 | 1.88 |  | 1.66 | | 1.00 | | 0.66 |
| **Noise** | 2.07 | 1.86 |  | 1.67 | | 0.74 | | 0.93 |
| **Music** | 2.18 | 2.05 |  | 1.90 | | 1.14 | | 0.76 |

**Table S7.** Classification accuracy of random forest (RF) connectivity-to-behaviour models (using development of language network to classify improvement of auditory and speech performance).

| **Condition** | Inter-hemisphere connections | |  | Intra-hemisphere connections | | | | |
| --- | --- | --- | --- | --- | --- | --- | --- | --- |
|  | Sum  (100) | Non-homo  (90) |  | Sum  (90) | Left intra (45) | | Right intra (45) | |
| **Resting state** | 82.97% | 79.81% |  | 83.48% | | 79.38% | | 84.75% |
| **Speech** | 77.56% | 77.24% |  | 73.38% | | 72.83% | | 76.65% |
| **Speech in noise** | 76.38% | 77.38% |  | 78.66% | | 79.30% | | 75.43% |
| **Noise** | 78.93% | 77.77% |  | 76.06% | | 73.92% | | 77.45% |
| **Music** | 76.92% | 78.14% |  | 77.01% | | 78.30% | | 73.58% |

**Table S8.** Classification accuracy of random forest (RF) connectivity-to-activity models (using development of language network at resting state and four types of stimulus conditions to classify development of left aTL responses to speech in noise).

| **Condition**  **predicting** | All  (190) | Inter  (90) | Intra  (90) | Left intra  (45) | Right intra  (45) |
| --- | --- | --- | --- | --- | --- |
| **Resting state** | 66.21% | 63.05% | 65.63% | 62.83%* | 70.08%* |
| **Speech** | 70.10% | 65.46% | 68.24% | 62.28%* | 66.34%* |
| **Speech in noise** | 61.88% | 60.78% | 60.43% | 63.08%* | 59.46%* |
| **Noise** | 67.22% | 71.93%* | 64.70%* | 63.48% | 65.00% |
| **Music** | 70.12% | 65.25% | 68.13% | 70.84%* | 64.38%* |

Red: t>10 compared with 4000 runs of permutation; *: At least one t>10 compared with permutation test & |t|>5 for independent two-sample *t* test. All, all connections; Inter, inter-hemisphere connections between non-homologous regions; Intra, within-hemisphere connections; aTL, anterior temporal lobe.

**Table S9.** Relative contributing weights of random forest (RF) connectivity-to-activity models (using development of language network at resting state and four types of stimulus conditions to classify development of left aTL responses to speech in noise).

| **Condition**  **predicting** | All  (190) | Inter  (90) | Intra  (90) | Left intra  (45) | Right intra  (45) |
| --- | --- | --- | --- | --- | --- |
| **Resting state** | 6.25 | 2.95 | 3.04 | 1.15 | 1.89 |
| **Speech** | 5.35 | 2.51 | 2.66 | 1.13 | 1.53 |
| **Speech in noise** | 4.43 | 2.22 | 2.10 | 1.24 | 0.86 |
| **Noise** | 5.16 | 2.51 | 2.50 | 1.10 | 1.40 |
| **Music** | 5.43 | 2.47 | 2.82 | 1.53 | 1.29 |

All, all connections; Inter, inter-hemisphere connections between non-homologous regions; Intra, within-hemisphere connections; aTL, anterior temporal lobe.

**Table S10.** Demographic and audiological information for 50 cochlear implanted (CI) children.

|  | **Number** | **Mean (SD)** |
| --- | --- | --- |
| **First test time after CI (mo.)** | / | 0.25 (0.29) |
| **Duration of two tests (mo.)** | / | 11.18 (9.65) |
| **Last test time after CI (mo.)** | / | 11.42 (9.72) |
| **Gender** |  |  |
| Female | 19 | / |
| Male | 31 |  |
| **CI side** |  |  |
| Left | 18 | / |
| Right | 31 |  |
| Both | 1 |  |
| **CIU (dB)** |  |  |
| <=90 | 4 | 103.94 (9.56) |
| 91-100 | 20 |  |
| 101-110 | 13 |  |
| >=111 | 13 |  |
| **NonCIU (dB)** |  |  |
| <=90 | 13 | 99.96 (13.47) |
| 91-100 | 13 |  |
| 101-110 | 13 |  |
| >=111 | 11 |  |
| **AoI (mo.)** |  |  |
| <=24 | 17 | 36.80 (18.26) |
| 25-36 | 12 |  |
| 37-48 | 11 |  |
| >=49 | 10 |  |
| **HAtime (mo.)** |  |  |
| <=6 | 10 | 14.84 (11.44) |
| 7-12 | 25 |  |
| 13-36 | 12 |  |
| >=37 | 3 |  |

CIU, residual hearing on CI side; Non-CIU, residual hearing on non-CI side; AoI, age of implantation; HAtime, duration of hearing aid.

**Table S11.** Montreal Neurological Institute (MNI) space of the 20 fNIRS channels.

|  |  | MNI-space | | | |
| --- | --- | --- | --- | --- | --- |
| Cortical area | | x | y | z | Brodmann area |
| aTL | L | -66.52 | -4.60 | -16.29 | 21 |
| aAC | L | -68.21 | -16.56 | 1.16 | 22 |
| mMTG | L | -70.15 | -28.55 | -10.29 | 21 |
| pAC | L | -68.68 | -40.86 | 10.60 | 22 |
| pMTG | L | -63.67 | -57.54 | -1.44 | 37 |
| AG | L | -57.39 | -62.76 | 20.83 | 39 |
| IPL | L | -62.80 | -47.78 | 37.36 | 40 |
| SMG | L | -67.85 | -25.86 | 26.22 | 40 |
| vMC | L | -65.22 | -4.16 | 20.93 | 4 |
| POp | L | -60.50 | 12.60 | 10.35 | 44 |
| aTL | R | 64.81 | -3.02 | -18.65 | 21 |
| aAC | R | 68.96 | -12.20 | -1.38 | 22 |
| mMTG | R | 70.34 | -26.72 | -10.66 | 21 |
| pAC | R | 68.89 | -37.02 | 10.64 | 22 |
| pMTG | R | 61.86 | -54.30 | -1.22 | 37 |
| AG | R | 56.35 | -59.15 | 23.49 | 39 |
| IPL | R | 61.30 | -44.42 | 38.27 | 40 |
| SMG | R | 68.50 | -23.45 | 26.68 | 40 |
| vMC | R | 63.75 | -1.25 | 19.40 | 4 |
| POp | R | 57.77 | 16.23 | 11.39 | 44 |

aTL, anterior temporal lobe; aAC, anterior auditory cortex; mMTG, middle middle temporal gyrus; pAC, posterior auditory cortex; pMTG, posterior middle temporal gyrus; AG, angular gyrus; IPL, inferior parietal lobule; SMG, supramarginal gyrus; vMC, ventral motor cortex; POp, pars opercularis of inferior frontal gyrus; L, left hemisphere; R, right hemisphere. Colour coding was consistent with Fig. 1a.
